# Targeting high risk forest goers for malaria elimination: a novel approach for investigating forest malaria to inform program intervention in Vietnam

**DOI:** 10.1186/s12879-020-05476-8

**Published:** 2020-10-15

**Authors:** Sara E. Canavati, Gerard C. Kelly, Cesia E. Quintero, Thuan Huu Vo, Long Khanh Tran, Thang Duc Ngo, Duong Thanh Tran, Kimberly A. Edgel, Nicholas J. Martin

**Affiliations:** 1 Vysnova Partners, Inc, 8400 Corporate Drive, Suite 130, Landover, MD 20875 USA; 2 Burnet Institute, 85 Commercial Road, Melbourne, VIC 3004 Australia; 3 National Institute of Malariology, Parasitology and Entomology, 34 Trung Van, Hanoi, 00000 Vietnam; 4 U.S. Naval Medical Research Unit TWO, PSA Sembawang Deptford Rd, Building 7-4, Singapore, 759657 Singapore

**Keywords:** Malaria elimination, Mobile and migrant populations, Forest malaria, Greater Mekong subregion, Vietnam

## Abstract

**Background:**

Individuals that work and sleep in remote forest and farm locations in the Greater Mekong Subregion continue to remain at high risk of both acquiring and transmitting malaria. These difficult-to-access population groups largely fall outside the reach of traditional village-centered interventions, presenting operational challenges for malaria programs. In Vietnam, over 60% of malaria cases are thought to be individuals who sleep in forests or on farms. New malaria elimination strategies are needed in countries where mobile and migrant workers frequently sleep outside of their homes. The aim of this study was to apply targeted surveillance-response based investigative approaches to gather location-specific data on confirmed malaria cases, with an objective to identify associated malaria prevention, treatment and risk behaviors of individuals sleeping in remote forest and farms sites in Vietnam.

**Methods:**

A cross-sectional study using novel targeted reactive investigative approaches at remote area sleeping sites was conducted in three mountainous communes in Phu Yen province in 2016. Index cases were defined as individuals routinely sleeping in forests or farms who had tested positive for malaria. Index cases and non-infected neighbors from forest and farm huts within 500 m of the established sleeping locations of index cases were interviewed at their remote-area sleeping sites.

**Results:**

A total of 307 participants, 110 index cases and 197 neighbors, were enrolled. Among 93 participants who slept in the forest, index cases were more likely to make > 5 trips to the forest per year (prevalence odds ratio (POR) 7.41, 95% confidence interval (CI) 2.66–20.63), sleep in huts without walls (POR 44.00, 95% CI 13.05–148.33), sleep without mosquito nets (POR 2.95, 95% CI 1.26–6.92), and work after dark (POR 5.48, 95% CI 1.84–16.35). Of the 204 farm-based respondents, a significantly higher proportion of index cases were involved in non-farming activities (logging) (POR 2.74, 95% CI 1.27–5.91).

**Conclusion:**

Investigative approaches employed in this study allowed for the effective recruitment and characterization of high-priority individuals frequently sleeping in remote forest and farm locations, providing relevant population and site-specific data that decision makers can use to design and implement targeted interventions to support malaria elimination.

## Background

Malaria remains a significant public health burden, with an estimated 228 million cases worldwide in 2018 [1]. To support efforts to eliminate this preventable disease, global bodies such as the World Health Organization (WHO) have published frameworks for malaria elimination to outline key pathways and priorities for countries pursuing these goals [2]. As individual malaria endemic countries refocus their national programs to align with an agenda of malaria elimination, decision-makers can face distinct operational challenges adapting global policies to local settings, where malaria transmission is often influenced by complex combinations of regional, country and sub-country specific factors.

Of global significance is the threat that increasing malaria drug resistance in the Greater Mekong Subregion (GMS) has to significantly impede and perhaps even reverse recent gains in malaria elimination efforts worldwide [3, 4]. At the regional level, key malaria elimination strategies in the GMS focus on strengthening country-led intervention combinations, with an emphasis on malaria case and entomological surveillance, monitoring and evaluation, and increasing access to essential services, particularly for the most vulnerable and at risk populations [5]. Together with multi-drug resistant malaria, the GMS faces additional region-specific challenges to malaria elimination including complex vector biology and forest malaria, an increasing prevalence of *Plasmodium vivax* (Pv) malaria, and large populations of vulnerable, mobile and remote individuals [3–6].

In line with global and regional goals, Vietnam is striving to eliminate malaria by 2030 [7, 8]. Key strategies adopted by the Vietnam National Institute of Malariology, Parasitology, and Entomology (NIMPE) to support elimination include improving access to early diagnosis and prompt effective treatment, ensuring uniform intervention coverage of at-risk populations, improving the malaria epidemiological surveillance system and ensuring sufficient capacity for malaria epidemic response [7]. To effectively combat malaria, Vietnam must address the significant challenges impacting the GMS, including forest malaria. The primary vector in Vietnam, *Anopheles dirus*, is an exophagic mosquito with recorded early evening biting behaviors that commonly inhabits forest, forest fringe and farm environments, and has been recorded in areas where large proportions of at-risk Vietnamese people work and sleep [9–11]. These complexities of malaria transmission may limit the effectiveness of traditional village-centric control interventions such as indoor residual spraying (IRS), insecticide treated nets (ITNs), and reactive case detection (RACD) focused around index case investigation and follow up at a village-based place of residence [9, 10].

In countries with large mobile and migrant populations (MMPs), such as Vietnam, a major limiting factor of village-focused RACD interventions is the location of exposure. Previous research shows that the majority of malaria infections occur among people who spend most of their nights sleeping outdoor either in the forest, forest fringes or on farms [10, 12]. It is estimated that over 60% of malaria cases reported in Vietnam are amongst individuals sleeping in forests or on farms [13]. Depending on occupation and other demographic characteristics, MMPs can spend days, weeks or months at a time in these vector-rich areas, sleeping in temporary and often communal housing structures, referred to as “huts”. In such settings, context specific and appropriately focused interventions are needed to attain malaria elimination [3, 11].

To support malaria elimination there is a need for operational research on forest-goers and the characterization of forest and farming activities in order to effectively target this high-priority population group with appropriate interventions [14]. Innovative approaches are required to capture relevant data at suspected malaria transmission forest and farm locations to better understand the micro-epidemiology and key risk factors in these remote yet critically important environments, and to support effective decision-making to implement context-specific actions. A novel approach was developed to implement targeted surveillance-response based investigative approaches at the point of suspected malaria transmission in three priority forested communes in central Vietnam. Aims of this study were to gather location-specific data relating to confirmed malaria cases where transmission was suspected to have occurred in forest or farm locations, and to identify associated malaria prevention, treatment and risk behaviors of individuals sleeping in these remote sites in Vietnam.

## Methods

### Site selection

**Fig. 1:**
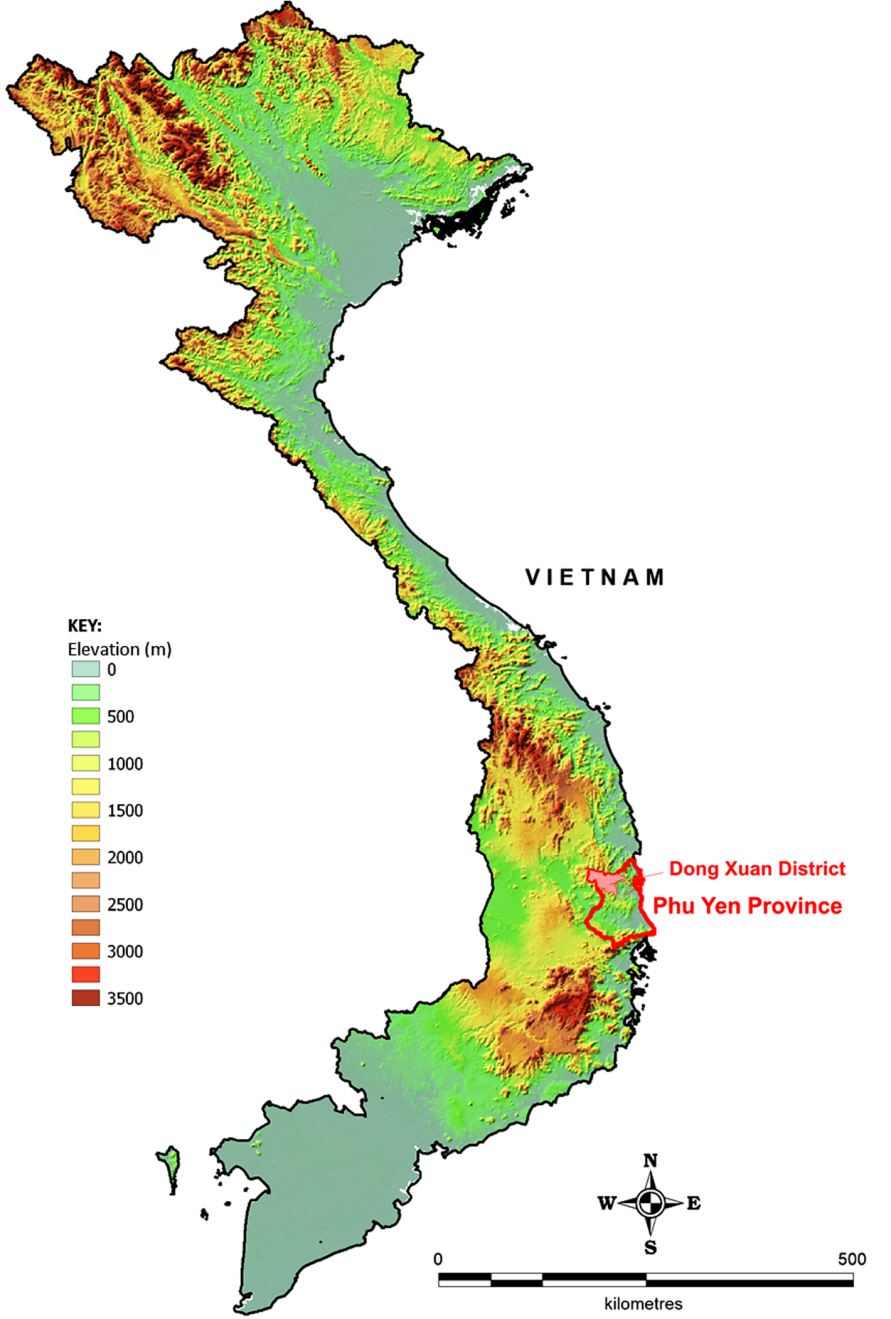
Study location site overlaid on digital elevation model data illustrating major mountain ranges in Vietnam. Custom map produced by the authors using *MapInfo Professional v15.0.2* (Pitney Bowes Software Inc. 2015, Stamford, CT; https://www.pitneybowes.com)

A cross-sectional study was conducted between April and October 2016 in three mountainous communes in Dong Xuan district, Phu Yen province: Phu Mo, Xuan Lanh, and Xuan Quang, in South Central Vietnam (Fig. 1). Sites were identified and selected by NIMPE and Phu Yen Department of Health Malaria Division due to their relative high malaria burden and high proportion of MMPs who frequently sleep in forests or forest fringe farms for their livelihood. These mountainous communes have also been deemed at high risk of drug resistant malaria, directly bordering two Tier 1 Artemisinin Resistant provinces (as defined by the WHO Global Plan for Artemisinin Resistance Containment [15]). Malaria transmission in Phu Yen displays strong seasonal patterns with bimodal peaks in transmission following the rainy seasons occurring in the Summer and Autumn months [16].

### Study design

Index case patient lists were acquired from Commune Health Centre’s (CHCs) within the study area, in accordance with national patient confidentiality guidelines, and a team of NIMPE and CHC staff were mobilized to interview these individuals at their respective remote area forest or farm site. At the remote area forest or farm site, all members of the two nearest neighboring huts or camp sleeping sites were invited to join the study. Each neighbor was asked to provide a single finger-prick blood sample for preparation of multispecies RDT (SD BIOLINE Malaria *Ag P.f/P.v*.). Neighboring individuals that were to test positive and suspected to have acquired malaria from a remote transmission site through a subsequent case investigation were to be recruited as an index case. Despite testing all neighbors for malaria infection however, no new infections were identified. Data were collected from index cases and neighbors at the remote area sleeping sites between September and October 2016. Index cases and neighbors were interviewed face-to-face using a structured questionnaire and observations made of the sleeping site. A total of 307 participants, 110 index cases and 197 neighbors, were included in the analysis. Ninety-three participants (54 index cases and 39 neighbors) slept exclusively in the forest and 204 participants (53 index cases and 151 neighbors) slept exclusively on farms. Ten participants slept in both forests and on farms and were excluded from forest and farm subgroup analysis.

### Study subject inclusion and exclusion

For the study, an index case was defined as an individual over 18 years of age who routinely slept in forest or farm locations, and had tested positive for malaria through passive case detection (PCD) via rapid diagnostic test (RDT) or microscopy at a CHC in the study area between 2014 and 2016. Additionally, index cases included only individuals where malaria transmission was suspected to have occurred in a forest or farm setting, classified as remote areas outside of urban or village settings. Suspected sites of malaria transmission were identified through case investigation reports and follow-up interviews with index cases upon recruitment. Patients clinically diagnosed with malaria without RDT or microscopy confirmation, confirmed malaria patients where transmission was suspected to have occurred within a village or residential setting, or patients unwilling to participate were excluded from the study.

Inclusion criteria for neighbors were: having tested negative for malaria by RDT or microscopy at the time of recruitment, residing in one of the two nearest huts to an index case hut or sleeping location within 500 m, currently sleeping at an accessible sleeping site, over the age of 18, and willing to participate in the study. Reasons for exclusion for neighbors were: having tested positive for malaria by RDT or microscopy between April and September 2016 or at the time of recruitment, having been clinically diagnosed without confirmation by diagnostic testing during those dates, having taken malaria prophylaxis or treatment in the 14 days preceding the study, and being unwilling or unavailable to participate. All eligible, willing and available index case and neighbor participants were included in the study.

### Data collection and analysis

Data collected included characteristics of sleeping site huts, travel duration from huts to nearest area of cellphone coverage, number of huts within a 500-m radius, type of water source and distance from hut, and mosquito usage. Additional information on hut members was also collected including demographics, occupation, previous malaria history, and malaria prevention knowledge and practices. All responses to questions were self-reported. Mosquito net ownership was also directly observed. Global position system (GPS) coordinates were also taken at each interview site.

The structured questionnaire was developed in English with input from NIMPE staff and translated into Vietnamese. Where necessary, ethnic minority language interviews were conducted in the appropriate language and translated into Vietnamese. The questionnaire was pre-tested in different communes of the district and revised accordingly. All members of the study team were trained in standardized interviewing techniques to obtain consistent data. Data were collected by NIMPE and CHC staff using a smartphone-based application (KLL Collect, Kathmandu Living Labs, Kathmandu, Nepal) and uploaded in real-time to a web-based data portal (Ona Systems, Nairobi, Kenya). Data were processed daily to ensure completeness and internal consistency. NIMPE personnel provided training to CHC staff on study procedures. All data collection forms were checked by field supervisors.

Data were analyzed using R software (Epi, car and BMA packages, version 3.1.4 R Foundation). Frequencies and proportions were used to describe categorical variables, and means were used to describe continuous variables. Chi square test was used to assess statistically significant differences in socio-demographic characteristics between the index cases and neighbors. Student’s t-test was used to assess the difference in mean age between study groups (index cases and neighbors). Shapiro-Wilk test was used to test the normality of data prior to selecting the measure of central tendency for continuous variables and applying Student’s t-test. Multivariable logistic regression models were used to calculate prevalence odds ratios (PORs) and 95% confidence interval (CI) to assess differences in characteristics among index cases and neighbors, after adjusting for socio-demographic characteristics (age, gender, ethnicity and education). Selection and goodness-of-fit of the models were assessed by Pearson chi square and deviance statistics. Effect size *Cramer’s V* or *Φ* were used to estimate the magnitude of the difference between categorical variables, and effect size Cohen’s *d* was used to estimate the magnitude of the difference between the means of two groups. When analyzing the differences between index case and neighbor groups, all responses to multiple choice questions were treated as independent (exclusive), except when specifically noted. A *P*-value of < 0.05 was considered significant.

## Results

### Demographic comparison of index cases and neighbors

Of the 307 participants, 233 (75.9%) were male; among index cases, 90 (81.8%) were male, whereas among neighbors, 143 (72.6%) were male (*P* = 0.070). The mean age was significantly higher (Student t test, two-tailed for independent samples, *P* < 0.001, ES Cohen *d* = − 0.35) among neighbors (mean 41.7 years, *SD* 14.8 years) than among index cases (mean 36.6 years, *SD* 13.4; Table 1).

**Table 1:** Socio-demographic characteristics among index cases and neighbors sleeping in at-risk sites of malaria, Phu Yen province, Vietnam, 2016

Characteristics	Category	Index Case	Neighbors	Total	***p***-value
Total		***n*** = 110	***n*** = 197	***n*** = 307	
Age (year, mean ± SD)		36.6 ± 13.4	41.7 ± 14.8	39.8 ± 14.5	< 0.001
Gender (n, %)	Male	90 (81.8)	143 (72.6)	233 (75.9)	0.070
	Female	20 (18.2)	54 (27.4)	74 (24.1)	
Ethnicity (n, %)	Kinh	52 (47.3)	70 (35.5)	122 (39.7)	0.044
	Cham	58 (52.7)	127 (64.5)	185 (60.3)	
Education levels (n, %)	Illiterate	25 (22.7)	60 (30.5)	85 (27.7)	0.147
	Primary school	32 (29.1)	63 (32.0)	95 (30.9)	0.600
	Secondary school	23 (20.9)	31 (15.7)	54 (17.6)	0.254
	High school or above	30 (27.3)	43 (21.8)	73 (23.8)	0.283
**Sleeping in the forest only**		***n = 54***	***n = 39***	***n = 93***	
Age (year, mean ± SD)		34.5 ± 10.5	40.5 ± 12.8	37 ± 11.8	< 0.01
Gender (n, %)	Male	51 (94.4)	35 (89.7)	86 (92.5)	0.396
	Female	3 (5.6)	4 (10.3)	7 (7.5)	
Ethnicity (n, %)	Kinh	40 (74.1)	35 (89.7)	75 (80.7)	0.059
	Cham	14 (25.9)	4 (10.3)	18 (19.3)	
Education levels (n, %)	Illiterate	5 (9.2)	–	5 (5.3)	
	Primary school	13 (24.1)	9 (23.1)	22 (22.7)	0.911
	Secondary school	17 (31.5)	5 (12.8)	22 (22.7)	0.037
	High school or above	19 (35.2)	25 (64.1)	44 (47.3)	0.006
**Sleeping on the farm only**		***n = 53***	***n = 151***	***n = 204***	
Age (year, mean ± SD)		35.8 ± 16.3	43 ± 14.3	41.2 ± 15.2	0.003
Gender (n, %)	Male	36 (67.9)	101 (66.9)	137 (67.2)	0.89
	Female	17 (32.1)	50 (33.1)	67 (32.8)	
Ethnicity (n, %)	Kinh	10 (18.9)	32 (21.2)	42 (20.6)	0.719
	Cham	43 (81.1)	119 (78.8)	162 (79.4)	
Education levels (n, %)	Illiterate	19 (35.8)	60 (39.7)	79 (38.7)	0.617
	Primary school	19 (35.8)	50 (33.1)	69 (33.8)	0.717
	Secondary school	6 (11.4)	25 (16.6)	31 (15.2)	0.361
	High school or above	9 (17)	16 (10.6)	25 (12.3)	0.223

*SD* Standard deviation

All participants belonged to either the Cham (185; 60.3%) or Kinh (122; 39.7%) ethnic groups. The proportion of Cham participants was significantly higher among neighbors (127; 64.5%) than among index cases (58; 52.7%), although small effect size (ES) indicated weak association between ethnicity and study group (index case or neighbor) (χ^2^ (*df* = 1; *n* = 307) = 4.06, *P* = 0.04, ES Φ = 0.12). The difference in proportions of self-reported illiterate index cases (25; 22.7%) and neighbors (60; 30.5%) was not statistically significant (χ^2^ (*df* = 1; n = 307) = 2.11, *P* = 0.147). The proportions of those who had graduated from secondary school was not significantly different between the index cases (23; 20.9%) and neighbors (31, 15.7%) (χ^2^ (*df* = 1; *n* = 307) = 1.30, *P* = 0.254) (Table 1).

### Occupation and location of index cases

**Fig. 2:**
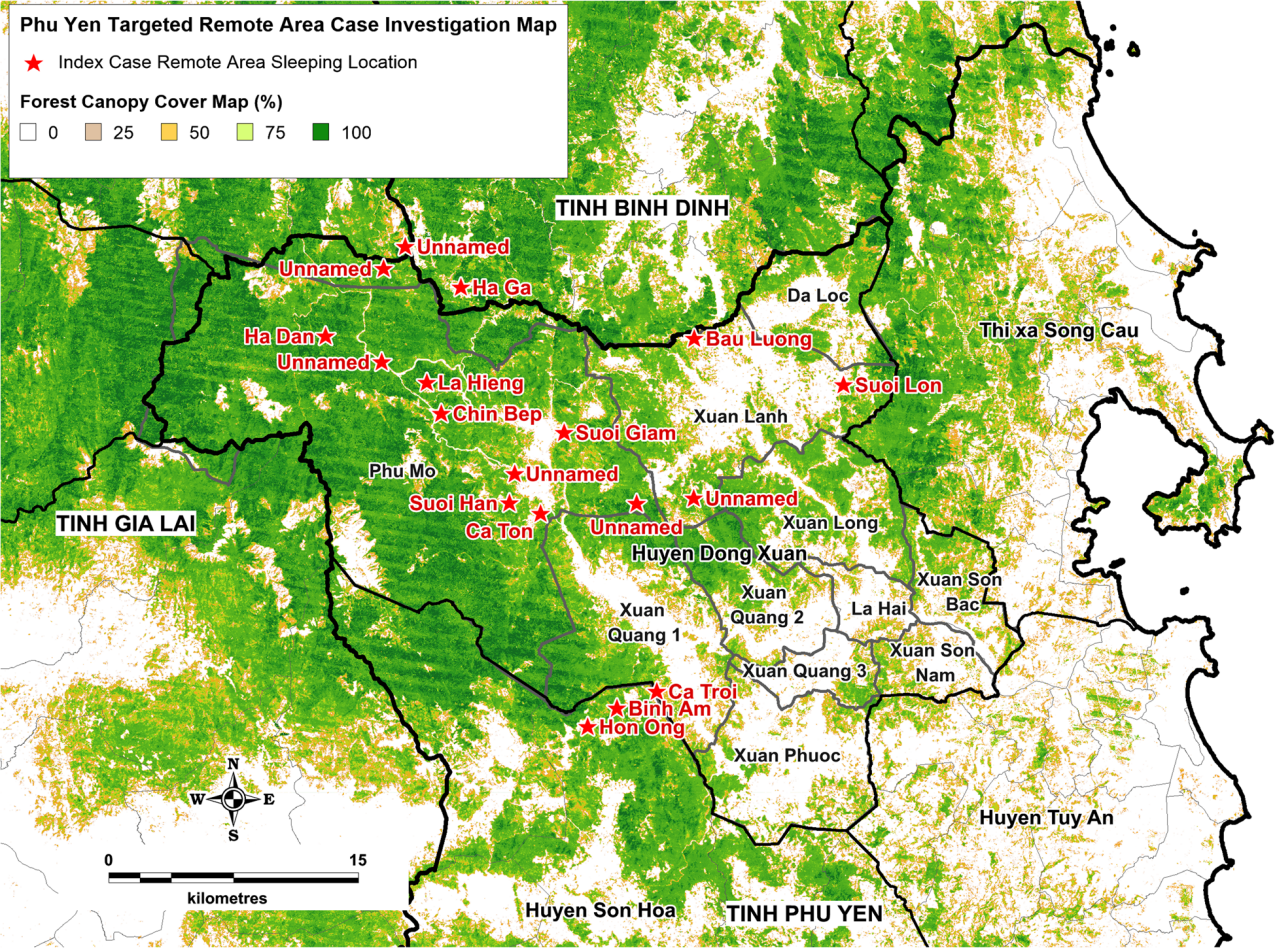
Index case remote area forest and forest fringe sleeping site locations map*. Custom map produced by the authors using *MapInfo Professional v15.0.2* (Pitney Bowes Software Inc. 2015, Stamford, CT; https://www.pitneybowes.com) (*tree cover canopy percentage spatial layer adapted from 30 m resolution global land cover satellite imagery spatial modelling data [27])

Index cases reported employment in harvesting aloe (27) or timber (5), farming cassava (24) or rice (19), trapping (2), plantation labor (16), construction (10), and other unspecified activities (7). Thirty-one (28%) index cases reported work activities at night, defined as between 6 pm and 5 am. A majority of index cases (59; 54%) originated from 14 specifically named remote area locations. The most common remote area locations were Ca Ton (13 cases; 12%) and Hon Ong (10 cases; 9%), while 51 (46%) were in unnamed locations. Within the 7–14 days before the onset of fever, just under half of the index cases were residing in either Ha Dan (25; 23%), Ca Ton (16; 15%), or Hon Ong (12; 11%). Figure 2 illustrates a map of index case remote area sleeping site locations and associated forest canopy cover.

### Forest demographics

Of the 93 respondents who slept in the forest only, 75 (80.7%) were of Kinh ethnicity while 40 (74.1%) of index cases were of Kinh ethnicity. All self-reported illiterate forest workers were index cases. There were significantly more neighbors with high school education or above than index cases (χ^2^ (*df* = 1; *n* = 93) = 7.60, *P* = 0.006, ES Φ = 0.29) (Table 1). Those who made more than five trips a year to the forest were 7.41 times more likely to be an index case (95% CI 2.66–20.63). Index cases were also more likely to sleep in huts without walls or in hammocks without a building structure (prevalence odds ratio (POR) 44.00, 95% CI 13.05–148.33). Additionally, index cases were more likely than neighbors to work in deep forest occupations, such as aloe collecting and trapping, (POR 11.67, 95% CI 4.37–31.18) and to work after dark (POR 5.48, 95% CI 1.84–16.35) (Table 2).

**Table 2:** Characteristics of index cases and neighbors sleeping in the forest and farm in Phu Yen province, Vietnam, 2016

Characteristics	Category	Index Case	Neighbor	Total	POR (95% CI)
***Sleeping in the forest***		***n = 54 (n, %)***	***n = 39 (n, %)***	***n = 93 (n, %)***	
Bed or hammock net use *more than one answer possible	- Do not use bed-nets or hammock nets -Use untreated nets *Adjusted for education level* -Use treated bed-nets *Adjusted for education level*	35 (64.8) 20 (37.0) 2 (3.7)	15 (38.5) 24 (61.5) 7 (17.9)	50 (53.8) 44 (47.3) 9 (9.7)	2.95 (1.26, 6.92) 0.37 (0.16, 0.86) 0.42 (0.17, 1.00)^a^ 0.18 (0.03, 0.90) 0.10 (0.02, 0.58)^a^
Forest trips per year	> 5 trips 5 trips or less	31 (57.4) 23 (42.6)	6 (15.4) 33 (84.6)	37 (39.8) 56 (60.2)	7.41 (2.66, 20.63)
Nights per trip in the forest	7 nights or less > 7 nights	27 (50) 27 (50)	3 (7.7) 36 (92.3)	30 (32.3) 63 (67.7)	12.00 (3.29, 43.72)
Type of place for sleeping	Hut without wall/outdoor Hut with wall	48 (88.9) 6 (11.1)	6 (15.4) 33 (84.6)	54 (58.1) 39 (41.9)	44.00 (13.05, 148.33)
Main work in the forest	Deep Forest: Aloe hunter, trapper, plantation and timber Forest Fringe: Forest guard, trader, construction worker	42 (77.8) 12 (22.2)	9 (23.1) 30 (76.9)	51 (54.8) 42 (45.2)	11.67 (4.37, 31.18)
Work after dark	Never Sometimes	49 (90.7) 5 (9.3)	25 (64.4) 14 (35.9)	74 (79.6) 19 (20.4)	5.48 (1.84, 16.35)
***Sleeping in the farm***		***n = 53***	***n = 151***	***n = 204***	
Bed or hammock net use	- Do not use bed-nets or hammock nets - Use untreated nets *Adjusted for education level* - Use treated bed-nets *Adjusted for education level*	23 (43.4) 21 (39.6) 13 (24.5)	67 (44.4) 50 (33.1) 56 (37.1)	90 (44.1) 71 (34.8) 69 (33.8)	0.96 (0.51, 1.81) 1.33 (0.69, 2.53) 0.55 (0.27, 1.12)
Forest trips per year	> 5 trips 5 trips or less	37 (69.8) 16 (30.2)	87 (57.6) 64 (42.4)	124 (60.8) 80 (39.2)	1.70 (0.85, 3.32)
Nights per trip in the forest	7 nights or less > 7 nights	36 (67.9) 17 (32.1)	95 (62.9) 56 (37.1)	131 (64.2) 73 (35.8)	1.25 (0.87, 2.43)
Type of place for sleeping	Hut without wall/outdoor Hut with wall	37 (69.8) 16 (30.2)	88 (53.3) 63 (41.7)	125 (61.3) 79 (38.7)	1.66 (0.85, 3.21)
Main work in the forest	Deep Forest: Aloe hunter, trapper, plantation and timber Forest Fringe: Forest guard, trader, construction worker	15 (28.3) 38 (71.7)	19 (12.6) 132 (87.4)	34 (16.7) 170 (83.3)	2.74 (1.27, 5.91)
Work after dark	Never Sometimes	26 (49.1) 27 (50.9)	60 (39.7) 91 (60.3)	86 (42.2) 118 (57.8)	1.46 (0.78, 2.73)

*POR* prevalence odds ratio, *CI* confidence interval, ^a^adjusted prevalence odds ratio

### Farm demographics

Of the 204 respondents who slept on farms only, 162 (79.4%) were of Cham ethnicity. Thirty-nine percent of both index cases and neighbors self-identified as illiterate; there were no statistical differences between index cases and neighbors regarding ethnicity and education. The majority of both index cases and neighbors made four or fewer trips per year to work at a farm (POR 1.70, 95% CI 0.85–3.32), and stayed for less than 20 days at a time (POR 1.25, 95% CI 0.87–2.43). Although both groups were predominantly employed in farming activities, index cases were more likely than neighbors to be employed in non-farming activities, such as logging (POR 2.74, 95% CI 1.27–5.91). There was no significant difference in the likelihood of working after dark for farm-based index cases and neighbors (POR 1.46, 95% CI 0.78–2.74) (Table 2).

### Mosquito net usage, ownership and attitudes

More than half of forest-based participants (50 cases; 54%) reported that they did not use a bed or hammock net on a regular basis, while 47% reported use of untreated bed nets and only 10% reported use of insecticide-treated nets (ITNs). There may be overlap in responses as respondents were given the option to select multiple answers for this question. Forest index cases were more likely than neighbors to sleep without any kind of net (POR 2.95, 95% CI 1.26–6.92) and less likely to use ITNs (POR 0.10, 95% CI 0.02–0.58). The difference in use of untreated bed nets between index cases and neighbors was significant in univariate analysis, but not significant after adjusting for education level (Table 2). Just under half of farm-based participants (90; 44%) reported that they did not use a bed or hammock net on a regular basis. Thirty-five percent and 34% used untreated nets and ITNs, respectively. The likelihoods of not using bed nets, using untreated nets and using treated nets were not significantly different for neighbors and index cases (POR 0.96, 95% CI 0.51–1.81, POR 1.33, 95% CI 0.69–2.53 and POR 0.55, 95% CI 0.27–1.12, respectively).

Observations and data on net ownership as well as attitudes were also captured in the study (Table 3). Approximately half (151; 49.2%) of all participants owned untreated nets and 113 (36.8%) owned treated nets. When asked, 121 (39.4%) of all participants indicated they were willing to use untreated nets at sleeping sites in the future: comprising 40 (36.4%) index cases and 81 (41.1%) neighbors. Participants owned 54 treated nets, of which index cases represented only 13 while neighbors accounted for 41 nets. Only 19 (10%) huts had treated nets: 4 (6%) index huts and 15 (14%) neighbor huts. When asked about willingness to use treated nets at sleeping sites only 40 (13%) participants indicated they were willing to use treated nets: 10 (9%) index cases and 30 (15%) neighbors. Only the difference in number of huts having untreated nets between index cases and neighbors was statistically significant (POR 0.40, 95% CI 0.20–0.78).

**Table 3:** Observations on using nets and attitude toward using nets in future among index cases and neighbors sleeping in at-risk sites of malaria, Phu Yen province, Vietnam, 2016

Observation at investigation time	Index cases (***n*** = 110) (n, %)	Neighbors (***n*** = 197) (n, %)	Total (***n*** = 307) (n, %)	POR (95% CI)
Own bed-nets or hammock with a net - Own untreated nets^a^ -Own treated nets^a^	57 (51.8) 33 (30.0)	94 (47.7) 80 (40.6)	151 (49.2) 113 (36.8)	1.18 (0.74, 1.88) 0.63 (0.38, 1.03)
Total of untreated nets in total huts	89	174	263	
Number of huts had untreated nets (*n* = 180)	17/71 (23.9)	48/109 (44.0)	65/180 (36.1)	0.40 (0.20, 0.78)
Willing to use untreated nets at sleeping sites	40 (36.4)	81 (41.1)	121 (39.4)	0.82 (0.51, 1.32)
Total of treated nets in total huts	13	41	54	
Number of huts had treated nets (*n* = 180)	4/71 (5.6)	15/109 (13.8)	19/180 (10.6)	0.38 (0.10, 1.13)
Willing to use treated nets at sleeping sites	10 (9.1)	30 (15.2)	40 (13.0)	0.56 (0.26, 1.19)

*POR* prevalence odds ratio, *CI* confidence interval

^a^Multiple responses possible

### Sleeping sites

Participants lived in 180 huts, of which 21 huts had two or more cases per hut and accounted for 60 (56%) index malaria cases, 50 huts had one case, and 109 huts had no cases. Index case huts had an average of 1.6 (SD = 1.1) members per hut. The 109 neighbor huts had an average of 1.8 (SD = 1.2) members per hut. Significantly higher number of index huts than neighbor huts had more than three occupants (POR 4.63, 95% CI 2.74–7.81); 97 (31.7%) of huts slept more than one person (minimum number) and 79 (25.8%) of huts slept more than five people (maximum number). The minimum and maximum number of people usually sleeping in a hut was significantly higher among the index huts than the neighbor huts, POR 3.23, 95% CI 1.96–5.34 and POR 4.15, 95% CI 2.42–7.10, correspondingly (Table 4).

**Table 4:** Characteristics of huts and activities among index cases and neighbors sleeping in at-risk sites of malaria, Phu Yen province, Vietnam, 2016

Characteristics	Category	Index case (***n*** = 110) (n, %)	Neighbors (***n*** = 197) (n, %)	Total (***n*** = 307) (n, %)	POR (95% CI)
Year huts were established	1996–2006 2007–2016	9 (8.2) 101 (91.8)	24 (12.2) 173 (87.8)	33 (10.7) 274 (89.3)	**0.64 (0.29, 1.44)**
Duration from home to huts by motorbike Additional time by walking	> 30 min 30 min or less > 15 min 15 min or less	51 (46.4) 59 (53.6) 40 (36.4) 70 (63.6)	43 (21.8) 154 (78.2) 48 (24.4) 149 (75.6)	94 (30.6) 213 (69.4) 88 (28.7) 219 (71.3)	**3.10 (1.87, 5.12)** **1.77 (1.07, 2.94)**
Number of people usually sleep in this hut in the last 12 months	> 3 persons 3 or less	55 (50.0) 55 (50.0)	35 (17.8) 162 (82.2)	90 (29.3) 217 (70.7)	**4.63 (2.74, 7.81)**
Minimum number of people used to sleep *(from 2 or more* vs *1 person)*		53 (48.2)	44 (22.4)	97 (31.7)	**3.23 (1.96, 5.33)**
Maximum number of people used to sleep *(from > 5* vs *5 persons or less)*		48 (43.6)	31 (15.8)	79 (25.8)	**4.15 (2.42, 7.10)**
Number of huts within 500 m radius of participants	> 3 huts 0–3 huts	23 (20.9) 87 (79.1)	19 (9.6) 178 (90.4)	42 (13.7) 269 (86.3)	**2.48 (1.29, 4.76)**
Number of people within 500 m radius of participants	> 6 neighbors 0–6 neighbors	22 (20.0) 88 (80.9)	22 (11.2) 175 (88.8)	44 (14.3) 263 (85.7)	**1.99 (1.04, 3.79)**
Duration from hut to nearest cell phone coverage by motorcycle	Network available 30 min or less 30–120 min	61 (55.5) 18 (16.4) 31 (28.2)	162 (82.2) 22 (11.2) 13 (6.6)	223 (72.6) 40 (13.0) 44 (14.3)	**0.27 (0.16, 0.45)** **1.56 (0.80, 3.03)** **5.55 (2.79, 11.07)**
Drinking water sources usually be used	Stream Well	95 (86.4) 15 (13.6)	136 (69.0) 61 (31.0)	231 (75.2) 76 (24.8)	**2.84 (1.54, 5.26)**

*POR* prevalence odds ratio, *CI* confidence interval

Two hundred and seventy-four (89.3%) participants lived in huts established in the last 10 years (from 2007 to 2016), including 101 (91.8%) of index cases and 173 (87.8%) of neighbors. Two hundred and thirteen (69.4%) of participants were able to reach their huts from their reported residence within 30 min by motorbike. Neighbors had huts significantly closer to their official homes than the index cases; neighbors’ huts were 3.10 times more likely to be within a 30-min motorbike ride (95% CI 1.87–5.13) and 1.77 times more likely to be within a 15-min of additional walking (95% CI 1.07–2.94) than index’s huts.

Two hundred and twenty-three (72.6%) of participants had immediate access to a cell phone network and 40 (13.0%) had access to a cell phone network within 30 min by motorbike. Access to a cell phone network was significantly lower among the index huts for immediate network (POR 0.27, 95% CI 0.16–0.45), but no significant difference was observed for network coverage within 30 min by motorbike (POR 1.56, 95% CI 0.80–3.03). About three quarters of the participants retrieved their water from a stream and the remaining quarter from a well. The odds of index cases taking water from a stream was 2.84, 95% CI 1.54–5.26) times higher than that of neighbors (Table 4).

## Discussion

Obstacles to malaria elimination remain not only for Vietnam, but the GMS as a region due to individuals working and sleeping in remote forest and forest fringe locations. This study presents a unique approach to investigate and characterize malaria behavioral risk factors in such remote areas of central Vietnam. There are significant challenges to identifying exposure sites for infection, associated risk factors and the transmission potential of malaria among MMPs. Such challenges are likely to limit the effectiveness of traditional village-centric malaria transmission reduction interventions in remote forest and forest fringe farm settings in the GMS, having clear operational impacts for malaria programs pursuing an elimination agenda. Conducting the study on-site in these remote locations guaranteed that all study participants were MMPs, unlike previous studies conducted in villages where a portion of the participants may not have worked in the forest or forest fringe areas known to be hotspots for malaria transmission [17]. Executing these studies in remote areas where malaria transmission is suspected to have occurred not only captures information of an individual’s behavior but also provides specific evidence-based data on site characteristics including remote living and sleeping conditions. Such data have traditionally been unavailable to program managers responsible for implementing local interventions. Given the challenges associated with accessing and collecting information on high-risk MMPs and their associated sleeping environments, opportunities exist to utilize these data to support the management of other priority infectious diseases impacting these population groups exist also.

Findings from this study indicate that both forest and farm index cases exhibited malaria risk behavior. From an operational perspective, these data provide practical information that can support the effective design and implementation of locally appropriate targeted interventions. Forest index cases made trips to forests more frequently each year than neighbors and were more likely than neighbors to sleep in huts without walls, sleep without bed nets, and work deep in the forest and after dark. This indicates these individuals spend longer periods of time without protective measures in remote areas receptive to malaria transmission. Farm index cases were also more likely than neighbors to be involved in forest-based activities, such as logging. Given the similarities of occupations in both forest and farm index cases, further efforts should be directed into identifying and targeting malaria preventions interventions that seek to mitigate malaria transmission risk when undertaking such activities in both settings. As malaria prevalence among MMPs was positively correlated with increased risk behavior, frequency of forest trips and involvement in deep-forest activities, these factors could be used as a surrogate for malaria exposure intensity. Such data identifies and describes a critical population sub-group where traditional malaria interventions are generally not reaching or protecting, and potentially may not suffice, highlighting a need for decision makers to develop and target alternative, appropriate and acceptable intervention measures.

Thirty percent of index case huts had multiple index cases; and index case huts had significantly more visitors than neighboring huts. Not only were there clustering of cases at remote area sleeping sites, but significantly more index huts than neighbor huts had more than three occupants. In addition to neighbor huts being less densely populated, they were also located significantly closer to the occupants’ reported residences than index case huts. This suggests that the neighbor hut occupants may have spent less time at these sites, with therefore less potential exposure time, since their work sites were more easily accessible to their individual homes. Conversely, index huts were further away from their reported residences, suggesting the occupants may have spent more time in remote area forest sites overall and therefore had a greater length of time at risk of exposure. These data suggest that the greater number of people sleeping in the same place and involved in forest activities, and staying for longer periods of time in remote areas, creates a more favorable setting for ongoing malaria transmission.

Although personal protective materials, such as long lasting insecticidal hammocks (LLIHs) and ITNs are available and effective, [18, 19], it is evident from this and other studies that their utilization remains challenging in remote areas [20]. Results from this study support previous findings that net usage is low among those sleeping in the forest [10, 21]. The proportion of index huts with either treated or untreated nets was lower than that of neighbor huts. Net use, even when supply was adequate, was especially poor among forest workers, where a strong negative association existed between net use and malaria infection. Alarmingly, the results indicated that only a small percentage of participants (13%) were willing to use ITNs at the sleeping sites. These findings present a significant operational challenge for decision makers in these regions, particularly if access to malaria prevention resources is limited to the more traditional interventions such as ITNs. These results suggest at-risk populations stand to benefit from tailored, appropriate and effective education and behavior change communication interventions. More targeted and novel approaches may be required to educate, engage and improve awareness among high risk MMPs of the importance of using available protective measures and the role they can play in disrupting ongoing malaria transmission. Additionally, a need also exists for program managers to understand the potential underlying factors behind any lack of willingness to use ITNs in the forest, and perhaps explore alternative personal protection options that may be more practically utilized and accepted in these settings. Examples could include the use of repellents or insecticide treated clothing, however such options would require further research and validation. Further investigation is needed to understand reasons behind the reluctance to use nets; identify additional strategies to effectively implement appropriate protective measures, including the implementation of appropriate public health education interventions to support increased update; and to ensure universal access and adoption of these measures can be assured for at-risk populations.

A majority of survey respondents were male, which is consistent with other studies [22, 23]. It is interesting to note that although an ethnic minority at the national level, Cham was the majority ethnic group captured in this study, indicating relatively higher proportions of Cham among MMPs within the general population of the area. Additionally, while there was relatively few Cham forest workers, 78% were index cases, indicating they are an important sub-group to target for malaria prevention. In this study, all self-reported illiterate respondents belonged to the Cham ethnic group. While not specifically investigated in this study, the high prevalence of self-reported illiteracy could be associated with lower understanding of malaria transmission and prevention, and may explain the overrepresentation of Cham people among the index cases. Neighbors were comprised of 9% more females than the index cases; this could indicate that women are engaged in jobs that do not expose them to malaria at the sleeping sites as much as their male counterparts. However, further research exploring gender roles in these remote sites and the relationship to high risk behavior is needed for more definite understanding of gender as a risk factor.

In the context of malaria elimination where total cases are relatively low, recruitment of a sufficiently large sample to identify statistically significant malaria risk factors is challenging. In Southeast Asia, barriers to locating shrinking malaria reservoirs are compounded by the fact that priority populations tend to be MMPs who are usually highly mobile, and priority areas are typically remote forest and peripheral locations that are difficult to access [6, 24, 25]. Approaches employed in this study leveraged passive detection systems, which led to a more efficient recruitment of both index case and neighbor study participants belonging to the same MMP subgroup than would have been possible through traditional field-based active case finding methods. Given the relatively love malaria prevalence in the GMS a much larger RACD study would likely be needed to uncover undiagnosed cases by RDT or microscopy. RACD studies are labor intensive and costly and there are decreasing resources available for malaria elimination programs [26].

Some limitations associated with logistical challenges operating in remote settings are noted. Not all huts within the vicinity of the index cases were able to be included in due to restricted access, and some index cases did not have any neighboring huts within a 500 m radius. It is also possible some cases may have been missed in extremely remote parts of the forest within the study area, introducing potential bias towards more accessible regions. Neighbors were screened using only RDT, with no capacity to take into account cases of low-level parasitemia that are only detectable by lab-based molecular testing such as polymerase chain reaction (PCR). Participants were asked to self-report information related to previous malaria exposure, time spent in the forest and other potential risk factors; as such there was a risk of recall bias for the information collected during interviews in this study.

## Conclusion

The unique targeted reactive investigative approach employed in this study allowed for the effective identification, recruitment and characterization of forest and farm going MMP participants. These approaches allow malaria programs to capture relevant population and site specific data that decision makers can use to design and implement locally appropriate interventions to address and build on macro-policy global and regional elimination strategies. Additional research is required to further evaluate the feasibility, impact and effectiveness of adopting targeted investigative approaches at scale within routine malaria program activities to characterize and support the implementation of focused interventions that directly address at-risk populations in remote transmission sites.

## Data Availability

The datasets generated during and/or analyzed during the current study are available from the corresponding author on reasonable request.

## Abbreviations

POR: Prevalence odds ratio
CI: Confidence interval
ITN: Insecticide treated net
WHO: World Health Organization
GMS: Greater Mekong Subregion
NIMPE: Vietnam National Institute of Malariology, Parasitology, and Entomology
IRS: Indoor residual spraying
RACD: Reactive case detection
MMP: Mobile and migrant population
NAMRU-2: Naval Medical Research Unit-TWO
PCD: Passive case detection
CHC: Commune Health Centre
RDT: Rapid diagnostic test
GPS: Global position system
LLIH: Long lasting insecticidal hammock
